# The utility of simple questions to evaluate cognitive impairment

**DOI:** 10.1371/journal.pone.0233225

**Published:** 2020-05-14

**Authors:** Yugaku Daté, Daisuke Sugiyama, Hajime Tabuchi, Naho Saito, Mika Konishi, Yoko Eguchi, Yuki Momota, Takahito Yoshizaki, Kyoko Mashima, Masaru Mimura, Jin Nakahara, Daisuke Ito

**Affiliations:** 1 Departments of Neurology, Keio University School of Medicine, Shinjuku-ku, Tokyo, Japan; 2 Faculty of Nursing and Medical Care Graduate School of Health Management, Keio University, Kanagawa, Japan; 3 Departments of Neuropsychiatry, Keio University School of Medicine, Shinjuku-ku, Tokyo, Japan; Niigata University, JAPAN

## Abstract

**Objectives:**

As the population of patients with cognitive decline grows, physicians and caregivers need brief screening tools. Comprehensive neurocognitive batteries require special training and time for evaluation. We focused on accessibility and compared the diagnostic power of several easy questions.

**Design:**

“Attended With” (AW) and “Head-Turning Sign” (HTS) factors and participants’ replies to following questions were recorded: “Do you feel that you have more difficulties in your daily life than you used to?”, [no consciousness (C-) or consciousness+ (C+)], “Could you tell me about your daily pleasures or pastimes?” [no pleasure (P-) or pleasure + (P+)], “What are notable current/recent news/topics?” [no news (N-) or news+ (N+)].

**Setting:**

This took place in our Memory Clinic between May 2016 and July 2019.

**Participants:**

We enrolled 162 consecutive cases (44 cognitive normal (CN), 55 amnestic mild cognitive impairment (aMCI), and 48 Alzheimer’s disease (AD)).

**Measurements:**

The sensitivity and specificity of each battery were calculated, and on account of those numbers, the population attributable risk percent % (PAR%) of (AW and HTS+), (C- and P-), (C- and N-), (P- and N-) as analysis of combination of questions, respectively, were calculated.

**Results:**

AW had high sensitivity, 87.4, 95.8% (CN vs aMCI + AD, CN + aMCI vs AD) but the sensitivity of HTS was only 46.4, 57.7%, and HTS showed high specificity, 100.0, 71.8%. C- had high sensitivity, 80.6, 87.5%, whereas P- and N- had high specificity, both 83.9% in CN vs aMCI + AD, 88.1% and 75.9% in CN + aMCI vs AD, respectively. In combination analysis, the PAR% of (C- and N-) were as high as (AW and HTS+).

**Conclusions:**

The combination of (C- and N-) is as powerful as (AW and HTS+) in screening AD. Our findings provide novel insights for screening utility of brief questions “Consciousness of Impairment” and “Recent News.”

## Introduction

The rate of memory loss is 41% among people aged 55–64 years and 52% among those aged 70–85 years [1]. As the worldwide population grows older at an unprecedented rate, complaints of memory loss and cognitive disorder will be more and more common [2–5]. In order to diagnose dementia accurately, a thorough medical history, physical and neurological examination, and neurocognitive assessment are needed [6]. Neurocognitive function should be assessed by means of comprehensive set of batteries [7], but these batteries require special training and much time for assessment that many clinicians are unable to spare. The Mini Mental State Examination (MMSE) is a brief examination to detect dementia, but it requires as long as ten minutes per patient and is reported to have low sensitivity, that is, a high rate of false negatives [8].

As more and more people develop cognitive dysfunction and most of them are not conscious of the fact that they are developing an illness [9, 10], prompt access to suitable medical care relies on primary physicians or caregivers who consult neurologists or psychiatrists, and more time-saving and easy assessment screening batteries are necessary. “Attended With (AW)” and “Head-Turning Sign (HTS)” have been reported to be the most effective set of screening batteries [11]. A patient with negative AW, which means he or she visits the clinic alone, is likely to have an absence of cognitive decline [12]. HTS, which is a patient's behavior of looking back to his or her accompanying person for help when asked something by the examiner, is reported to be a strong marker of dementia [13]. Soysal P. *et al*. reported the effectiveness of AW and HTS as a marker of cognitive decline, especially focusing on older populations who are most likely to develop dementia [14].

But there are some limitations to these batteries. AW has high sensitivity to detect cognitive decline in older adults [14] but cannot evaluate the cognitive function of hospitalized patients. To identify HTS, at least one accompanying person is necessary, so is not always suitable for hospitalized patients or those who live alone. In cases where a professional caregiver wants to evaluate a client’s cognitive function in the client’s home, neither AW nor HTS is an effective tool. Further, both are affected by cultural settings and regional/national characteristics. In searching for easy screening questions for cognitive impairment, we focused on briefness, effectiveness (sensitivity and specificity), and accessibility to all older adults.

## Materials and methods

### Participants

Between May 2016 and July 2019, 162 consecutive participants, aged 60 to 89 years at the first medical examination, were recruited from the memory clinic of Keio University Hospital as a retrospective clinical cohort. The study design and protocol were approved by the Ethics Committee for Human Research of the Keio University School of Medicine (#20170315). Informed consent was obtained in the form of opt-out on the web-site (https://www.neurology.med.keio.ac.jp/newslist/). Those who rejected were excluded.

### Assessment

All subjects underwent thorough medical history, neurological examination, and serum examination. After that they underwent at least two types of imaging studies, including head magnetic resonance imaging (MRI) and cerebral fluid single photon emission computed tomography (SPECT), and if needed, myocardial ^123^I-Metaiodobenzylguanidine (MIBG) scintigraphy and / or dopamine transporter scan. They were also examined with conclusive neurocognitive batteries including the MMSE, Logical Memory subtest of Wechsler Memory Scale revised (LM), Rey-Osterrieth Complex Figure Test (ROCFT), Ray Auditory Verbal Learning Test (RAVLT), Trail Making Test (TMT), Verbal Fluency Test (initial letter and category), Neuropsychiatric Inventory—Questionnaire (NPI-Q), Zarit Burden Interview, GDS and Geriatric Depression Scale15 (GDS15) for diagnosis, as previously described [7]. For RAVLT, the five learning trials on category A list (trials 1–5) were followed by an interference category B list, followed by two post-list B interference recall trials, an immediate and delayed, then a recognition trial with distractor words [15].

Diagnoses of dementia were assigned after several visits by dementia specialists according to reference criteria [16–19].

### Brief questions

On a participant’s first visit to our memory clinic, AW and HTS were recorded. AW was based on whether the patient first visited the clinic accompanied by someone else, such as a family, friend, or caretaker. The accompanying person was instructed to sit next to or behind the patient at an angle of 45°, and if the patient turned his or her head at least one time seeking for help during the medical interview, he or she was recorded as “HTS+” [14].

We asked every patient three questions: “Do you feel that you have more difficulties in your daily life than you used to?”; “Could you tell me about your daily pleasures or pastimes?”; and “What are notable current/recent news/topics?” In this study, all subjects were patients visiting our hospital, not a community-based population; the majority of patients visiting alone had some memory or cognitive-related complaints. Thus if a patient answered, “Yes, I have difficulties in my daily life,” they were classified as self-aware [“Consciousness + (C+)”]; if they answered, “No, I do not have difficulties”, they were classified as having “unconsciousness, anosognosia, (C-)” in the recorded results. Only when a patient provided a specific and concrete answer like “I enjoy feeding our dog and going for a walk to a nearby park with him” was the patient recorded as engaging in “Pleasure or pastime+ (P+)”. When the patient answered nothing, or the answer was subtle or abstract (like “Generally I enjoy everything”), the patient was recorded as “(P-)”. When a patient provided an example of concrete, adequate, recent news (within half a year), he or she was recorded as aware of “news+ (N+)”, but if the patient answered nothing, or gave an abstract answer like “There have been a number of recent events”, or provided news more than half a year old, he or she was recorded as “no news (N-)”.

### Data analysis

For these five types of batteries, AW, HTS, Consciousness, Pleasure or Pastime, and Recent News, the number of patients diagnosed as Alzheimer’s disease (AD), amnestic mild cognitive impairment (aMCI), and as cognitively normal were counted. In this step, the diagnosis of dementia only included dementia caused by AD spectrum (AD, mixed dementia (AD and VD, AD and DLB, aMCI). Other forms of dementia were excluded. The sensitivity and specificity to distinguish cognitive normal (CN) + aMCI vs dementia, and CN vs aMCI + dementia, were calculated respectively for these five batteries. To clarify which combination of questions is the most impact for diagnosis, we estimated the attributable risk percentage (PAR%), [1− (1/odd ratio)]*100%, of (AW and HTS+), (C- and P-), (C- and N-), and (P- and N-), each for CN + aMCI vs AD, and CN vs aMCI + AD, respectively. Significant differences among groups for each neuropsychiatric battery were examined by the Student’s t test.

## Results

### Participants

The characteristics of 162 patients who visited the Memory Clinic in Keio University Hospital and their final diagnoses are demonstrated in Table 1. In terms of educational history, 45.7% (74 out of 162) patients had 16 years of education or more, much higher than the 19.9% average percentage of university graduates in the Japanese population[20].

**Table 1:** Characteristics and diagnoses of patients.

	Age(years old)	Gender (female/male)	Education (years)	MMSE (mean±SD)	GDS (mean±SD)
CN (44)	70.6±13.8	1.20(24/20)	15.0±1.9	28.3±1.7(n = 43)	3.4±3.3(n = 39)
aMCI(55)	79.6±7.1*	1.39(32/23)	13.5±2.6*	25.8±1.2*(n = 53)	4.1±3.7(n = 52)
AD(48)	80.6±6.8*	2.00(32/16)	12.8±2.7*	20.0±3.6*^#^(n = 44)	4.0±3.7(n = 46)

*: P<0.05 (CN vs aMCI + AD),

^#^: P<0.05 (aMCI vs AD)

Significant differences among groups for each value were examined by the Student’s t test

MMSE was recorded in 154 patients with an average score of 24.45±4.30. One hundred fifty-one out of 162 patients underwent GDS15, and the average score was 4.00±3.74. Out of 162 patients, 48 patients were diagnosed as AD, all 55 MCI were diagnosed as aMCI, and 44 were CN, including subjective cognitive impairment. Fifteen residual patients were diagnosed either as dementia with DLB, FTLD, epilepsy after encephalitis, chronic alcohol addiction, or iNPH. We focused on 44 CN and 103 patients with cognitive impairment due to AD spectrum [AD including mixed dementia (AD and VD, DLB and AD), aMCI.].

### Sensitivity and specificity of each clinical sign/question

The number of patients who were classified as CN, aMCI, or AD in each clinical sign are shown in Table 2. Notably, 42 patients were visiting alone (AA), of whom only two were AD. No CN was HTS positive and only 10 subjects with CN and aMCI showed P-.

**Table 2:** Numbers of patients classified as NC, aMCI or AD in each clinical sign.

	Attend		HTS		C		P		N	
	AA	AW	AWxHTS-	AWxHTS+	C+	C-	P+	P-	N+	N-
CN(44)	65.9%(29)	34.1%(15)	100%(9)	0%(0)	68.2%(30)	31.8%(14)	83.9%(26)	16.1%(5)	83.9%(26)	16.1%(5)
aMCI(55)	20.0%(11)	80.0%(44)	63.3%(19)	36.7%(11)	25.5%(14)	74.5%(41)	90.6%(48)	9.4%(5)	71.25(37)	28.8%(15)
AD(48)	4.2%(2)	95.8%(46)	42.3%(11)	57.7%(15)	12.5%(6)	87.5%(42)	72.7%(33)	28.3%(13)	30.4%(14)	69.6%(32)

Table 3 shows the sensitivity and specificity of each sign to assess cognitive function. Both AW+ and C- show high sensitivity, more than 80%, in both CN vs aMCI + AD and CN + aMCI vs AD. The sensitivity of AW was higher than that of C- in both CN vs aMCI + AD and CN + aMCI vs AD. Both P- and N- had 83.9% specificity to distinguish the CN and aMCI + AD, and the specificity of P-, 88.1%, was the highest in distinguishing CN + aMCI vs AD. The highest specificity in distinguishing CN vs aMCI + AD was shown by HTS+, but in distinguishing CN + aMCI vs AD, the specificity of HTS+ was lower than P- or N-.

**Table 3:** Sensitivity and specificity of each sign in assessing cognitive function.

	AW	HTS+	C-	P-	N-
CN vs aMCI+AD sensitivity (%)	87.4%	46.4%	80.6%	18.2%	48.0%
CN vs aMCI+AD specificity (%)	65.9%	100.0%	68.2%	83.9%	83.9%
CN+aMCI vs AD sensitivity (%)	95.8%	57.7%	87.5%	28.3%	69.6%
CN+aMCI vs AD specificity (%)	40.4%	71.8%	44.4%	88.1%	75.9%

Taken together, sensitivity was highest in AW, followed by C- in both CN vs aMCI + AD and CN + aMCI vs AD, and the specificity was highest in HTS+ in CN vs aMCI +AD, but higher in P- or N- in CN + aMCI vs AD. We propose that all clinical signs and questions in this study have diagnostic significance for memory clinics.

### Analysis of combination of questions

The specificity of P- in CN + aMCI vs AD is higher than that of N-, but the absolute number of AD patients in P- group is smaller than that in C- or N- group, so the diagnostic power of C-, P-, and N- is not simply compared only by the sensitivity and specificity. To clarify which combination of questions is most effective for diagnosis, we analyzed population attributable risk % (PAR%), which is widely used to evaluate the estimated potential impact of clinical sings and/or risk factors, as shown in Table 4. To distinguish aMCI + AD from CN, the PAR% of each combination of signs group is 29.9% (AA and HTS+), 7.45% (C- and P-), 22.8% (C- and N-), 2.04% (P- and N-) respectively. To distinguish AD from CN or aMCI, the PAR% is 56.6% (AA and HTS+), 23.1% (C- and P-), 57.6% (C- and N-), and 17.6% (P- and N-), respectively. Collectively, the cognitive sign set, (AW and HTS+) and (C- and N-), most distinguish aMCI + AD from CN, and AD patients from the CN+aMCI, respectively.

**Table 4:** Population attributable risk % of each sign to assess cognitive function.

	AA		AW		
	-		HTS-	HTS+	HTS no record
n [CN]	29		9	0	6
n [aMCI]	11		19	11	14
n [AD]	2		11	15	20
OR(CN vs aMCI + AD)	0.31		0.74	1	-
**(AW and HTS+) PAR% (CN vs aMCI + AD)**		**29.90%**			
OR(CN + aMCI vs AD)	0.048		0.26	0.62	-
**(AW and HTS+) PAR% (CN + aMCI vs AD)**		**56.60%**			
	C+		C-		
	P+	P-		P+	P-
n [CN]	17	3		9	2
n [aMCI]	13	1		35	4
n [AD]	3	1		29	12
OR(CN vs aMCI + AD)	0.48	0.4		0.88	0.89
**(C- and P-) PAR% (CN vs aMCI + AD)**		**7.45%**			
OR(CN + aMCI vs AD)	0.09	0.2		0.4	0.67
**(C- and P-) PAR% (CN + aMCI vs AD)**		**23.10%**			
	C+		C-		
	N+	N-		N+	N-
n [CN]	16	4		10	1
n [aMCI]	11	3		26	12
n [AD]	1	4		13	28
OR(CN vs aMCI + AD)	0.43	0.64		0.8	0.98
**(C- and N-) PAR% (CN vs aMCI + AD)**		**22.80%**			
OR(CN + aMCI vs AD)	0.04	0.36		0.27	0.68
**(C- and N-) PAR% (CN + aMCI vs AD)**		**57.60%**			
	P+		P-		
	N+	N-		N+	N-
n [CN]	25	1		1	4
n [aMCI]	34	13		3	2
n [AD]	12	21		2	11
OR(CN vs aMCI + AD)	0.65	0.97		0.83	0.76
**(P- and N-) PAR% (CN vs aMCI + AD)**		**2.04%**			
OR(CN + aMCI vs AD)	0.17	0.6		0.33	0.65
**(P- and N-) PAR% (CN + aMCI vs AD)**		**17.60%**			

OR: odd ratio

### Each question and neuropsychiatric batteries

In Table 5, the mean value and standard deviation of a set of neuropsychiatric batteries in each question (C+, C-, P+, P-, N+, N-), and the P-value of Student’s t-test comparing the positive group and negative group in each sign are shown. N- has a consistent correlation with the memory batteries (RALVLT1, ROCFT3min, LMI) and executive function tests (TMTA, verbal fluency), but otherwise, even with NPI, Zarit, or GDS, no consistent correlation was observed. it was shown that C- and P- are independent parameters of neuropsychiatric batteries.

**Table 5:** Comparison among three signs (C-, P-, N-) and points in each neuropsychiatric battery.

	MMSE	educ. Years	NPI Severity	NPI Distress	Zarit	RCPM	RALVLT1	RALVLT5	RALVLTpost	RALVLTrec	RALVLTdis	
C+ (n = 50)	24.3±4.8	13.1±3.0	3.53±4.19	3.87±4.12	23.7±19.9	24.4±8.8	2.56±1.50	6.67±3.11	3.00±2.68	12.3±2.1	3.17±2.15	
C- (n = 97)	22.7±3.9	13.2±2.8	3.69±4.91	3.60±4.35	17.4±16.6	22.2±6.6	2.52±1.49	5.91±2.25	2.72±2.28	12.0±2.8	3.01±1.65	
p value	0.204	0.365	0.898	0.818	0.262	0.325	0.932	0.337	0.682	0.661	0.775	
P+ (n = 107)	23.2±4.0	12.7±3.0	3.79±5.15	3.79±4.60	17.9±16.9	23.1±6.8	2.60±1.57	6.08±2.27	2.74±2.37	12.1±2.7	3.20±1.79	
P- (n = 23)	22.5±4.0	13.5±1.9	3.05±3.20	2.90±2.90	17.5±13.4	19.5±7.2	2.16±1.12	5.74±3.12	3.00±2.33	12.1±2.6	2.37±1.38	
p value	0.542	0.265	0.424	0.288	0.91	0.061	0.16	0.653	0.67	0.994	0.033	
N+ (n = 77)	24.4±3.5	13.2±2.7	3.00±5.19	2.89±3.84	15.8±16.0	23.8±6.1	2.87±1.54	6.67±2.33	3.30±2.52	12.0±2.6	3.41±1.85	
N- (n = 52)	21.6±4.2	12.8±2.8	4.29±4.42	4.33±4.65	19.7±16.4	20.8±7.67	2.12±1.25	5.35±2.39	2.40±2.05	12.2±2.8	2.71±1.54	
p value	0.000†	0.418	0.183	0.078	0.213	0.034	0.007†	0.009	0.074	0.643	0.057	
	ROCFTcopy	ROCFT3min	LMI	LMII	TMTA	TMTB	StroopI	StroopII	StroopIII	WF I	WF C	GDS
C+ (n = 50)	33.5±3.5	10.1±8.2	4.53±2.93	1.68±1.97	157.7±66.7	233.5±128.1	23.3±13.2	29.3±14.8	41.3±23.0	19.2±9.8	27.5±9.8	4.93±3.97
C- (n = 97)	31.2±6.5	6.30±4.84	4.34±3.04	1.98±2.30	193.7±99.3	302.6±187.0	24.5±10.2	39.3±20.9	52.1±30.6	17.9±9.2	25.2±9.8	3.70±3.32
p value	0.038	0.073	0.801	0.57	0.062	0.124	0.709	0.021†	0.098	0.601	0.361	0.153
P+ (n = 107)	31.9±5.6	7.30±5.83	4.47±2.95	2.10±2.34	186.2±96.2	275.6±175.1	24.8±11.4	38.2±20.4	51.2±28.7	18.0±8.5	25.5±9.4	5.00±4.03
P- (n = 23)	29.9±8.6	5.34±4.84	3.79±3.19	0.95±1.27	199.7±94.8	375.2±191.7	22.9±6.5	36.6±20.5	48.8±35.1	17.5±12.5	25.2±10.0	4.40±4.10
p value	0.339	0.135	0.399	0.004†	0.589	0.152	0.33	0.758	0.784	0.87	0.88	0.033
N+ (n = 77)	32.7±4.5	8.46±6.01	5.00±3.01	2.31±2.37	164.9±80.4	281.0±186.8	24.5±11.3	34.7±16.0	44.7±23.3	19.8±9.6	28.0±8.3	4.54±3.96
N- (n = 52)	30.4±7.6	5.51±4.91	3.71±2.84	1.47±2.03	218.9±105.2	302.9±175.4	24.9±10.1	41.4±24.0	56.8±34.8	15.7±8.4	22.7±9.9	3.98±3.74
p value	0.076	0.008†	0.030†	0.055	0.006†	0.982	0.837	0.133	0.065	0.024†	0.004†	0.557

Significant differences among groups for each value were examined by the Student’s t test (†: p<0.05), MMSE: Mini-mental state examination, NPI: Neuropsychiatric Inventory, Zarit: Zarit Burden Interview, Raven’s Colored Progressive Matrices (RCPM), Logical Memory Subtest of Wechsler Memory Scale-Revised (LM), Rey Auditory Verbal Learning Test (RAVLT), Trial 1 (RALVLT1), Trial 5 (RALVLT5), post-list B interference recall trial (RALVLTpost), a recognition trial (RALVLTrec), distractor words (RALVLTdis), Rey-Osterrieth Complex Figure Test (ROCFT), copy trial (ROCFTcopy), a recall trial 3 min (ROCFT 3min), Modified Stroop test (Stroop), Trail Making Test (TMT), and Verbal Fluency (VF), GDS: Geriatric depression scale 15

## Discussion

We found AW and C- had high sensitivity, and P- and N- had high specificity for evaluating cognitive impairments. To evaluate the impact of question sets, PAR% of (C- and N-) were higher than that of (C- and P-) and that of (P- and N-), so (C- and N-) was considered to be the suitable tool to detect the AD without the presence of a family member or caregiver. Previous reports [11, 14] demonstrated that AW and HTS were practical, time-saving, and sensitive tools to detect patients’ cognitive impairments, but these tools could be applied only in cases where a patient has family or a caregiver. The cognitive function of a patient who has no family members or who is hospitalized for a physical disorder other than dementia and is suspected to have some cognitive disorder cannot be assessed with these tools. In this study we measured the power of several alternative tools to detect cognitive impairment of older adults.

In a previous report from Turkey [14], the sensitivity of HTS was 80.95% and the specificity 64.76%, which is remarkably higher than those in our study. The age distribution was 75.67±8.29 years in that study and is not significantly different compared our study, but the education years (7.43±4.72) were shorter than those in our study. The low sensitivity and high specificity of HTS in our study may have been caused by the high education level of the patients, which caused a feeling of self-respect and independence, or by cultural background, such as social status, relationships with spouse and children, family structure, and national character. Future studies should assess how differences in educational background affect the diagnostic utility of the tests used.

When interpreting the results of the present study, several limitations should be kept in mind. First, FTLD, VD and DLB patients were excluded from this study, so further investigation is needed to evaluate use of tests assessing frontal lobar functions to differentiate FTLD, VD, DLB and AD. Second, the CN group was basically composed of “cognitively preserved” normal persons, but this group included those with so-called “subjective cognitive impairment” [21]. As expected, C- is high prevalence, 31.8% in CN. We thought that it was practical to compare performance on our neuropsychological tests among the AD, aMCI, and CN groups in the outpatient clinic, but a future study should investigate a larger number of truly “normal” individuals. Third, patients with severe dementia were not included in the present study, presumably because they have difficulty in visiting a university hospital, and the significant difference in age and the educational history between CN, aMCI, and AD groups cannot be excluded. Fourth, subjects on the AD spectrum in this study were not evaluated by biomarkers of amyloid β burden, such as amyloid positron emission tomography and cerebrospinal fluid AD assessment. The selection and/or subdivision of participants by AD biomarkers will be fruitful for the interpretation of results in future studies. Fifth, the mean age and disease duration of our subjects were limited. Finally, our study may be subject to biases, including selection bias and information bias as a result of the retrospective study.

All our subjects were Japanese people living in an urban area under a generous welfare system, places where elderly people can live alone, even with dementia; therefore, further longitudinal studies of subjects of different races and ethnicities from various areas are necessary. Accessibility to clinics or hospitals and health insurance systems will also need to be considered in the interpretation of this study. Additionally, long-term follow-up of the cognitive function and assessment of clinical signs will also be helpful in determining the diagnostic significance of these clinical signs and questions; however, our findings provide novel insights for simple diagnostic tools.

## Conclusion

Our study suggests the combination of C- and N- are as powerful screening signs as AW and HTS. But on the other hand, our data suggest that the reactivity to these kinds of signs can differ based on cultural or educational background. Further worldwide data accumulation is needed for a comprehensive assessment of what kind of tools are most effective in assessing the cognitive function of patients.
